# Branched Actin Maintains Acetylated Microtubule Network in the Early Secretory Pathway

**DOI:** 10.3390/cells11010015

**Published:** 2021-12-22

**Authors:** Azumi Yoshimura, Stéphanie Miserey-Lenkei, Evelyne Coudrier, Bruno Goud

**Affiliations:** 1 Molecular Mechanisms of Intracellular Transport Laboratory, Institut Curie, PSL Research University, CNRS (Centre National de la Recherche Scientifique), UMR 144, 75005 Paris, France; azumi-y@yamaguchi-u.ac.jp (A.Y.); stephanie.miserey-lenkei@curie.fr (S.M.-L.); evelyne.coudrier@curie.fr (E.C.); 2 Science Research Center, Institute of Biomedical Research and Education, Yamaguchi University, Ube 755-8505, Japan

**Keywords:** membrane trafficking, Golgi apparatus, RAB1 GTPase, Arp2/3, microtubules

## Abstract

In the early secretory pathway, the delivery of anterograde cargoes from the endoplasmic reticulum (ER) exit sites (ERES) to the Golgi apparatus is a multi-step transport process occurring via the ER-Golgi intermediate compartment (IC, also called ERGIC). While the role microtubules in ER-to-Golgi transport has been well established, how the actin cytoskeleton contributes to this process remains poorly understood. Here, we report that Arp2/3 inhibition affects the network of acetylated microtubules around the Golgi and induces the accumulation of unusually long RAB1/GM130-positive carriers around the centrosome. These long carriers are less prone to reach the Golgi apparatus, and arrival of anterograde cargoes to the Golgi is decreased upon Arp2/3 inhibition. Our data suggest that Arp2/3-dependent actin polymerization maintains a stable network of acetylated microtubules, which ensures efficient cargo trafficking at the late stage of ER to Golgi transport.

## 1. Introduction

The implication of the actin cytoskeleton in the secretory pathway was historically provided by studies that reported perturbations in the kinetics of secretory protein traffic by actin toxins such as cytochalasin B, latrunculin and botulinum C2 [1,2,3]. Later on, many studies illustrated the presence of actin, actin regulators and effectors in the Golgi area [4,5,6,7,8]. It is now well established that the actin cytoskeleton plays essential roles in maintaining the Golgi architecture and supporting post-Golgi transport events. For instance, we showed that actomyosin contributes to the local rigidity of the Golgi complex and regulates its mechanics [9]. Actin polymerization, together with myosin motors, exert forces that actively drive membrane deformation and allow the fission of RAB6 positive transport carriers from *trans*-Golgi network (TGN) membranes [10]. Arp2/3-dependent actin polymerization and myosin 1b have been implicated in the budding of clathrin- and AP1-dependent carriers from TGN membranes [11,12,13,14]. In the pre-Golgi stage of secretory pathway, WHAMM (WASP homolog-associated protein with actin, membranes and microtubules) associates with membranes of the endoplasmic reticulum (ER)-Golgi intermediate compartment (IC) (also called ERGIC) and *cis*-Golgi membranes. WHAMM is recruited by RAB1, a small GTPase indispensable for ER-to-Golgi transport [15,16], which modulates its Arp2/3-dependent actin-nucleation activity [17,18]. Recently, actin filaments defined by tropomyosin Tpm4.2 have also been involved in ER to Golgi trafficking [19].

The IC/ERGIC compartment is a complex membrane system between ER exit sites (ERES) and the Golgi complex that spans the entire cytoplasm. It is commonly thought to emerge through clustering and/or fusion of cargo containing vesicles into vesiculotubular compartments in the vicinity of ERES [20,21,22,23,24,25,26,27,28,29]. Time-lapse imaging revealed the existence of distinct populations of ERGIC characterized by their protein composition and dynamics. Cargo receptors such as p58/ERGIC-53 and the KDEL receptors accumulate in stationary ERGIC structures and support post-ER sorting processes [30,31]. RAB1 exhibits an overlapping distribution with ERGIC-53 but also associates with fast-moving anterograde transport carriers [32,33]. Anterograde delivery of cargoes from ERES to the Golgi is not a single-step transport step. Time-lapse imaging of ERGIC-53, RAB1, and cargoes, such as temperature-sensitive mutant G protein from vesicular stomatitis virus (tsO45-VSV-G) revealed that dynamic elements move along microtubules in a stop-and-go fashion for long distances, and stop at multiple stationary elements [30,34,35,36]. In addition, the microtubule network has been shown to centralize a population of RAB1-positive (RAB1+) membranes in a compartment located around the centrosome, referred to as the pericentrosomal IC (pcIC) [36]. This pericentrosomal domain of the IC communicates with the Golgi apparatus via tubular and vesicular carriers and is in a close relationship with the endocytic recycling compartment (ERC) [36,37,38].

In this study, we sought to gain insight into whether and how Arp2/3-dependent actin polymerization contributes to the operation of the early secretory pathway. We focused on different steps of anterograde transport using time-lapse imaging and synchronized cargo release using the RUSH system [39]. We report that Arp2/3 inhibition affects the organization of acetylated microtubule network around the Golgi, and increased the accumulation of RAB1+ carriers around the centrosome. This led to decreased cargo transport to the Golgi apparatus, which suggests that efficient cargo trafficking during the last steps of ER-to-Golgi transport requires stable microtubule architecture supported by the actin cytoskeleton.

## 2. Materials and Methods

### 2.1. Cell Culture and Transfection

HeLa cells and HeLa cells stably expressing RUSH TNF𝛼 constructs (KDEL-Streptavidin and TNFα-SBP-EGFP) were cultured at 37 °C with 5% CO_2_ in DMEM (Gibco high glucose GlutaMAX™, Thermo Fisher Scientific, Waltham, MA, USA) supplemented with 10% FCS (Eurobio, Les Ulis, France) and 1 mM sodium pyruvate (Thermo Fisher Scientific).

Transfections with DNA plasmids of GFP-RAB1A (gift from Dr. Jaakko Saraste, University of Bergen, Bergen, Norway), mCherry-tubulin, and Centrin1-mCherry (gift from Dr. Manuel Théry, University of Paris, Paris, France) were performed using X-tremeGENE™ 9 (Roche, Basel, Switzerland) according to manufacturer’s instructions for 14–24 h. RUSH mannosidase II (ManII) constructs (Ii-Streptavidin and ManII-SBP-EGFP, gift from Dr. Gaelle Boncompain, Institut Curie, Paris, France) were transfected as previously described [39].

### 2.2. Chemical Treatment and Immunofluorescence

Cells were transferred to medium containing 25 mM HEPES and maintained at 37 °C for at least 1 h prior to chemical treatment. They were then treated at 37 °C with reagents indicated in the figure at following concentrations: 0.1% DMSO, 100 µM CK-666 (Sigma-Aldrich, St. Louis, MO, USA), 100 µM CK-689 (Millipore, Burlington, MA, USA), 2 µM Tubacin (Sigma-Aldrich). For CK-666 washout experiments, treated cells were transferred to fresh full medium at 37 °C.

For synchronized cargo release experiments, cells expressing RUSH constructs were treated with DMSO or CK-666 for 30 min at 37 °C, followed by addition of biotin at a final concentration of 40 µM. After 30 min, cells were fixed as described below.

For microtubule depolymerization, cells transfected with RUSH ManII constructs were maintained at 4 °C for 1.5 h and subsequently transferred to 37 °C medium containing 10 µM nocodazole. After 30 min, CK-666 or DMSO were added to the medium and incubated for 30 min, and finally biotin was added 30 min prior to fixation.

All cells were chemically fixed with 4% paraformaldehyde (Electron Microscopy Sciences, Hatfield, PA, USA) in NaHCa buffer (100 mM NaCl, 30 mM HEPES, and 2 mM CaCl_2_, pH 7.4) for 20–40 min at room temperature. For tubulin immunolabeling, cells were fixed and permeabilized with 100% methanol at −20 °C for 6 min. Fixed cells were washed with PBS, permeabilized with 0.05% saponin or 0.1% TritonX-100 for 15 min and incubated 1 h with primary antibodies against acetylated tubulin (clone 6-11B-1, Sigma-Aldrich), ERGIC-53 (a gift from Dr. Jaakko Saraste), ϒ-tubulin (a gift from Dr. Michel Bornens, Institut Curie, Paris, France), GMAP210 (a gift from Dr. Rosa Rios, Universidad de Sevilla-CSIC-Universidad Pablo de Olavide, Sevilla, Spain), giantin (Recombinant Antibody Platform, Institut Curie, Paris, France), GM130 (610823, BD Biosciences, Lake Franklin, NJ, USA), mannosidase IA2 (HPA034559, Sigma-Aldrich), mannosidase II (AB3712, Millipore), RAB1:GTP (Recombinant Antibody Platform, Institut Curie), RAB6:GTP (clone AA2, AdipoGen, San Diego, CA, USA), and TGN46 (AbD Serotec AHP500, BioRad, Hercules, CA, USA). Cells were then incubated 45 min with Alexa Fluor secondary antibodies (Jackson ImmunoResearch Laboratory, West Grove, PA, USA). Immunostained cells were imaged with a Nikon Inverted Eclipse Ti-E (Nikon, Tokyo, Japan) with a CSU-X1 spinning disk confocal unit (Yokogawa, Tokyo, Japan) equipped with a CoolSNAP HQ^2^ CCD camera (Photometrics, Tucson, AZ, USA) using CFI Plan Apo VC 60×/1.40 objective or with a Prime95B Scientific CMOS camera (Photometrics) using CFI Plan Apo 100×/1.40 objective.

### 2.3. Image Analysis of Chemically Fixed Cells

All images were processed and analyzed using ImageJ (U.S. National Institutes of Health, Bethesda, MD, USA). All intensity quantifications were performed after subtracting background intensity.

To estimate ManII-EGFP arrival to the Golgi after cargo release, percentage of EGFP intensity in the Golgi region was quantified. Cells transfected with RUSH constructs were fixed 30 min after cargo release and immunolabeled using Giantin and GM130 antibodies. Confocal Z-stacks were obtained at 0.3 µm step-size and sum intensity projections were obtained for each channel. The Golgi region was manually selected using Giantin or GM130 (excluding the tubular elements) labeling, and EGFP intensity in the Golgi region was quantified. Total EGFP intensity of a cell was quantified within the cell area manually defined from bright field images.

To estimate the acetylated tubulin level per cell, total intensity of anti-acetylated tubulin signal in each cell was quantified from sum intensity projections of confocal Z-stacks obtained at 0.3 µm step-size, and normalized to cell area estimated from Z projection.

### 2.4. Live-Cell Imaging and Analysis

Cells were cultured and transfected in phenol red-free medium and imaged at 37 °C in a 5% CO_2_ and humidified air chamber. Time-lapse imaging was performed with a Nikon Inverted Eclipse Ti-E with a Yokogawa CSU-X1 Spinning disk confocal unit equipped with a Prime95B Scientific CMOS camera (Photometrics) using CFI Plan Apo 100×/1.40 objective. Basal side of cells were imaged in all experiments.

The length of tubular GFP-RAB1+ elements before and after CK-666 addition (Figure 3B) were compared using time-lapse recording of a same cell. Motile tubular RAB1 elements were manually selected from equal number of frames from recordings before and 10–30 min after the addition of CK-666 and their length was quantified. To obtain relative distance of the selected RAB1 element from the Golgi (Figure 3C), a line between centroid of the Golgi and centroid of a RAB1 element was extended to the cell edge. Ratio of distance between the Golgi and RAB1 to the distance between the Golgi and cell edge was defined as relative distance and plotted against length of each carrier.

To obtain length of TNF𝛼-containing carriers during ER-to-Golgi transport, cells stably expressing RUSH TNF𝛼 constructs were treated with DMSO or CK-666 for 30 min prior to biotin addition. Confocal time-lapse imaging at 0.2 s/frame started ~2 min after cargo release. Length TNF𝛼 tubules were quantified from frames at 3 and 4 min after cargo release (Figure 5C). To obtain length of nascent TNF𝛼 carriers, stationary globular elements were chosen, and their length were measured from ≥3 consecutive frames after the onset of directional movement. The average value was used as length.

### 2.5. RAB1 Tracking

GFP-RAB1+ elements were manually tracked in the time lapse recordings obtained at 0.2 s/frame using the frames corresponding to 15–23 min after DMSO or CK-666 addition. RAB1 elements undergoing directional movement for at least ≥25 frames (5 s) were chosen, and tracking was performed using Manual Tracking plugin in ImageJ (https://biii.eu/manual-tracking-imagej (accessed on 3 May 2021)). During the ≥5 s tracking, RAB1+ elements moved in stop-and-go fashion. In our recordings, stationary globular RAB1+ elements exhibited saltatory movement at a speed up to 0.11 µm/frame, and therefore frames scored >0.11 µm/frame was defined as motile and otherwise as pause. Speed of individual RAB1+ element was determined from the mean value of all the ‘motile’ frames. To estimate how much RAB1+ carriers approached the destination, the Golgi, net movement of individual RAB1+ element to the Golgi centroid during 4-s tracking was calculated using manual tracking data.

### 2.6. Statistics

**Figure 1:**
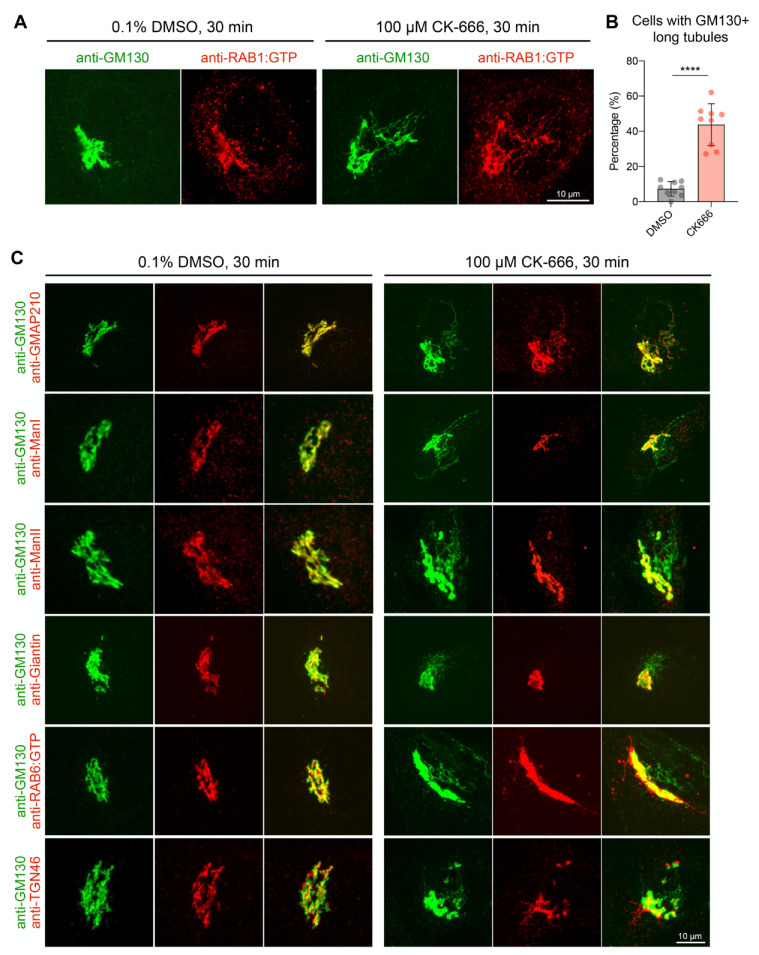
Arp2/3 inhibition induces an accumulation of GM130+ and RAB1+ membrane tubules. (**A**) HeLa cells treated with DMSO or CK-666 were fixed and immunolabeled with anti-GM130 and anti-RAB1:GTP antibodies. Maximum projections of confocal slices around the Golgi are shown. Scale bar: 10 µm. (**B**) Percentage of cells containing long GM130+ tubules. Raw data and mean ± SD are indicated (DMSO, *n* = 542; CK-666, *n* = 714 cells); **** *p* < 0.0001. (**C**) HeLa cells co-labeled with antibody against GM130 and antibodies against markers of *cis*-, medial, *trans*-Golgi, and TGN. Maximum projections of confocal slices around the Golgi are shown. Scale bar: 10 µm.

Data were statistically analyzed using GraphPad Prism (GraphPad Software; La Jolla, CA, USA). Data distributions were assumed to be normal for the following tests: unpaired *t* test with Welch’s correction for Figures 1B and 4C and Supplementary Figure S2B; one-way ANOVA Tukey’s multiple comparisons test for Supplementary Figure S1B and Figure 7E; and unpaired *t* test with or without Welch’s correction for Figure 4F,I. Data distribution was assessed by D’Agostino and Pearson test for the following tests. Mann Whitney test for Figures 3B, 5B,D,F, and 7B; and unpaired *t* test for Figures 3B and 6B. To assess the relationship between two variables, Spearman’s correlation test was used for Figure 3C, and simple linear regression for Figure 4. n.s., not significant; *, *p* < 0.05; **, *p* < 0.01; ***, *p* < 0.001; ****, *p* < 0.0001.

## 3. Results

### 3.1. Arp2/3 Inhibition Induces an Accumulation of RAB1+ Tubules around the Centrosome

To investigate the role of Arp2/3-dependent actin polymerization in the early secretory pathway, we first examined the morphology of the RAB1+ compartment in fixed HeLa cells treated with the Arp2/3 inhibitor CK-666. As shown in Figure 1A, CK-666 induced the appearance of long endogenous RAB1:GTP+ tubular structures containing the *cis*-Golgi matrix protein GM130. In control cells, RAB1+ elements can display a tubular aspect but over 95% of the tubules were less than 2.6 µm in length (Supplementary Figure S1A). We thus counted cells that contain tubules of ≥2.6 µm in CK-666-treated cells. As shown in Figure 1B, this was the case for about 50% of cells. Long tubules were not observed when cells were incubated with the inactive analog of CK-666, CK-689 (Supplementary Figure S1B). The CK-666 effect was also reversible as the tubular structures disappeared 30 min after removal of the drug (Supplementary Figure S1C; Supplementary Video S5).

In addition to RAB1, the GM130+ long tubular structures contained the *cis*-golgin GMAP-210 (Figure 1C). ERGIC-53 was also found in these structures but colocalization between ERGIC-53 and GMAP-210 was only partial, suggesting that only part of the IC is affected by CK-666 treatment (Supplementary Figure S1D). On the other hand, the CK-666 induced tubules did not contain the Golgi enzymes mannosidase I and II, nor the medial Golgi matrix protein giantin (Figure 1C). We noticed that CK-666 also induces a tubulation of TGN membranes defined by RAB6 and TGN-46. However, they are likely of different origin as RAB6+ and TGN-46+ tubules do not contain GM130 (Figure 1C). This effect of CK-666 on TGN membranes was not investigated further in this study.

**Figure 2:**
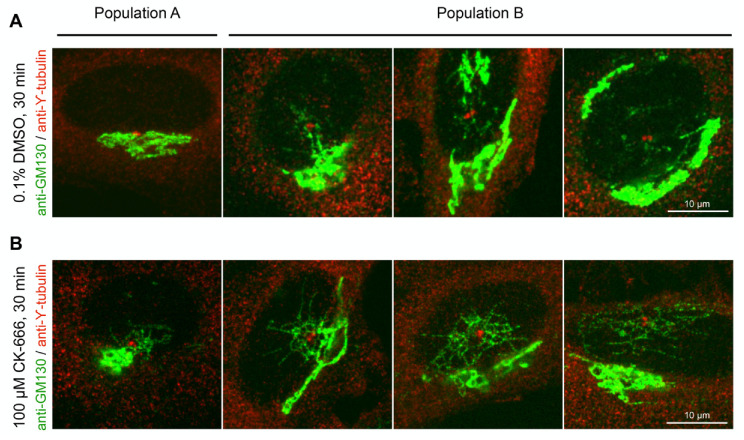
Emergence of CK-666-induced membrane tubules occurring around the centrosome. Cells were divided into two populations (**A**,**B**) based on the position of the centrosome relative to Golgi membranes. Cells treated with DMSO (**A**) or CK-666 (**B**) were fixed and immunolabeled with anti-GM130 and anti-γ-tubulin antibodies. Maximum projections of confocal slices around the Golgi from representative examples are shown. Scale bar: 10 µm.

We next investigated the position of the RAB1/GM130+ long tubules in relation to that of the centrosome. Cells were divided into two populations, one (population A) in which the centrosome (labelled with *γ*-tubulin) is embedded or closely affixed to Golgi membranes (labelled with GM130), and the other (population B) where the centrosome is localized away from Golgi membranes (Figure 2) [36]. The treatment with CK-666 did not change the relative percentage of each population (Supplementary Figure S2A). Very few RAB1/GM130+ long tubules were observed in control cells from population A (Figure 2A). After treatment with CK-666, they were observed in about 40% of cells (Figure 2B, Supplementary Figure S2B), a value close to the one found above (Figure 1B), consistent with the fact that the vast majority of cells belongs to population A. The majority of cells in population B already displayed long GM130+ tubules before treatment with CK-666 (Figure 2A, Supplementary Figure S2B). This percentage slightly increased after addition of the drug, and the majority of cells now showed prominent accumulation of RAB1/GM130+ long tubules around the centrosome (Figure 2B, Supplementary Figure S3B).

The above results suggest that CK-666-induced tubulation mainly takes place around the centrosome in the pcIC compartment. The lower percentage of tubules observed in population A is likely due to the difficulty of visualizing tubules when they are hidden by Golgi membranes.

### 3.2. Arp2/3 Inhibition Increases the Length of GFP-RAB1A+ Tubules at the Cell Center

**Figure 3:**
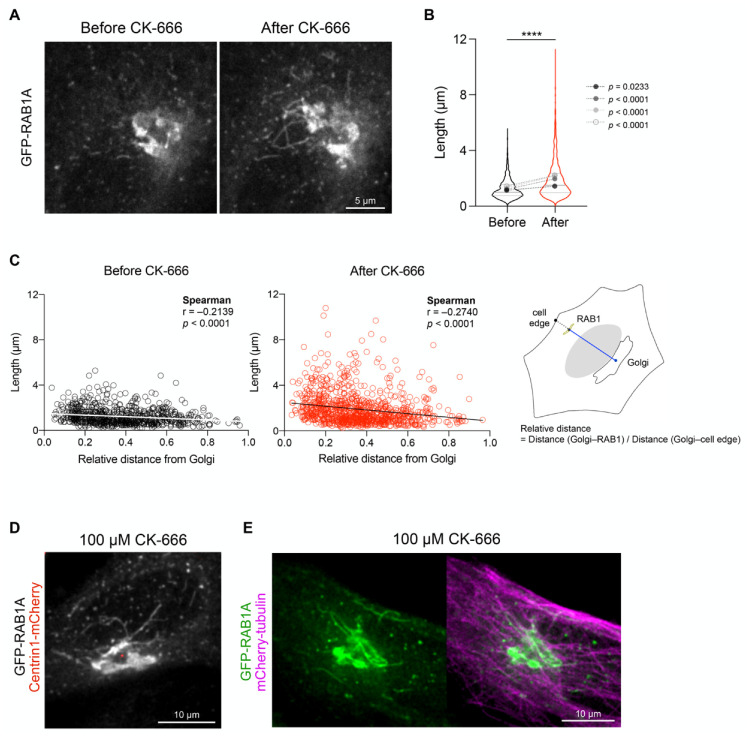
Arp2/3 inhibition increases the length of GFP-RAB1+ tubules at the cell center. (**A**–**C**) Length and position of GFP-RAB1+ tubules were monitored in GFP-RAB1 expressing HeLa cells before and after the addition of CK-666 by live-cell confocal microscopy. (**A**) Long GFP-RAB1+ tubules appear at the cell center after incubation with CK-666. GFP-RAB1+ tubules in the Golgi area of the same cell are shown from time-lapse recordings before and 20 min after CK-666 addition; Scale bar: 5 µm. (**B**) Quantification of the length of mobile GFP-RAB1+ tubules before and 10–30 min after the addition of CK-666. Violin plots of raw data (DMSO, *n* = 768; CK-666, *n* = 1002) from four cells are indicated with median and quartiles (lines). Mean (dots) and *p* values of individual cell are shown. **** *p* < 0.0001. (**C**) Length of GFP-RAB1+ tubules quantified in (**B**) plotted against their relative distance from the Golgi. Relative distance = distance (Golgi membranes–RAB1)/distance (Golgi membranes–cell edge). (**D**,**E**) HeLa cells expressing GFP-RAB1 together with centrin1-mCherry (**D**) or mCherry-tubulin (**E**) in the presence of CK-666; Scale bar: 10 µm.

To characterize the nature of the pericentrosomal RAB1+ tubules induced by Arp2/3 inhibition, we investigated their dynamics by time-lapse video-microscopy. Experiments were performed in cells expressing low levels of GFP-RAB1A in which GFP-RAB1A+ structures resembled those stained with the anti-RAB1:GTP antibody (endogenous RAB1). We first monitored changes in GFP-RAB1A before and after CK-666 addition. At steady state, GFP-RAB1A+ membranes appeared as dynamic pleomorphic structures (vesicles and short tubules) animated by bidirectional movement but with a tendency to move from the periphery to the cell center (Figure 3A; Supplementary Videos S1 and S2). Their average length was comparable to that found in DMSO-treated fixed cells (Supplementary Figure S1A). Following addition of CK-666, long GFP-RAB1A+ tubules started to appear around the Golgi and the centrosome after 10–15 min (Figure 3A,B,D; Supplementary Videos S1–S3). We quantified the length of GFP-RAB1+ tubules as a function of their position relative to Golgi membranes and the cell edge. As shown in Figure 3C, the longest ones appeared to be more concentrated in the cell center.

Dynamic GFP-RAB1+ tubules aligned along microtubules and showed extension/retraction movements (Figure 3E; Supplementary Video S4). Upon removal of CK-666, they continued to show bidirectional movements around the Golgi apparatus but progressively disappeared (Supplementary Video S5).

### 3.3. The Length of RAB1+ Tubules Correlates with Their Speed and Ability to Move towards the Golgi Apparatus

**Figure 4:**
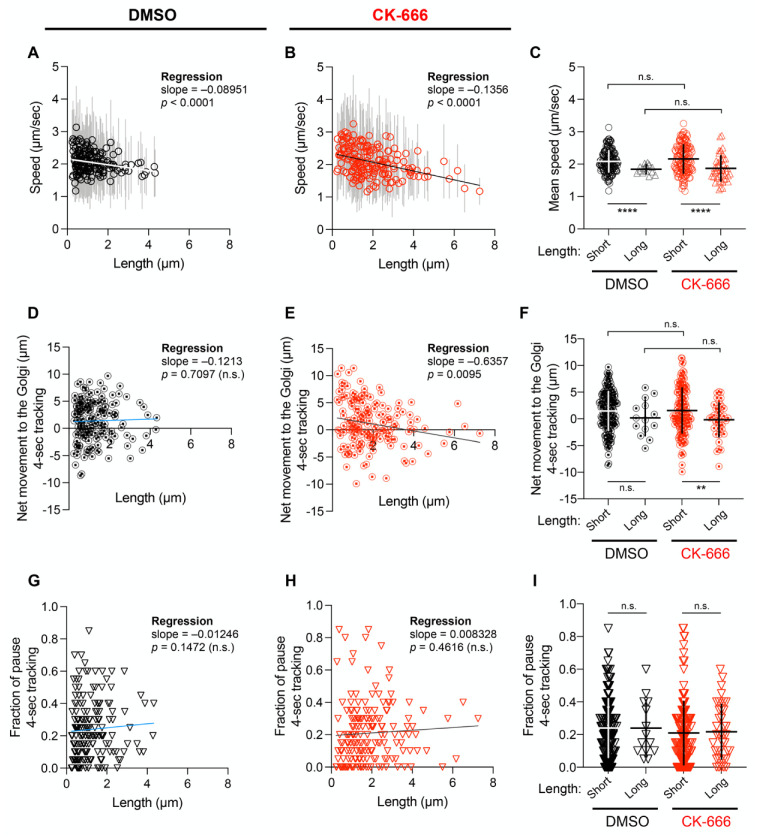
Tracking of GFP-RAB1+ tubules during directional movement from time-lapse confocal microscopy. Four cells were recorded. (**A**,**B**) Mean speed of individual GFP-RAB1+ element during the entire tracking was quantified and plotted against its length; mean ± SD and linear regression lines are indicated. (**C**) Mean speed values obtained in (**A**,**B**) are shown for short (<2.6 µm) and long (≥2.6 µm) GFP-RAB1+ tubules; the lines indicate mean ± SD of speed. (**D**,**E**) Distances that individual RAB1+ structure travels towards or away from Golgi membranes during 4-s tracking plotted against its length. Positive and negative values indicate that RAB1–Golgi distance decreases or increases, respectively, after 4-s. Raw data and linear regression lines are indicated. (**F**) Results categorized in a same manner as (**C**). (**G**,**H**) Pause was defined as frames ≤ 0.55 µm/s and fraction of pause during 4-s tracking was plotted against carrier length; raw data and linear regression lines are indicated. (**I**) Result categorized in a same manner as (**C**). n.s. not significant; ** *p* < 0.01; **** *p* < 0.0001.

We then investigated the relationship between the length of RAB1+ tubules and their speed using fast imaging (0.2 s intervals). We selected GFP-RAB1+ elements exhibiting directional movement for 4 s or longer and tracked them manually. The mean speed of short (<2.6 µm) or long (≥2.6 µm) tubules did not change after incubation with CK-666 (Figure 4B). However, the speed of long tubules was significantly lower than that of short tubules. This was confirmed by plotting the speed against length, which showed a negative correlation between the two parameters (Figure 4A,B).

We also evaluated whether a correlation exists between the tubule length and their ability to move towards the Golgi. For this purpose, we measured the distance between RAB1+ tubules and Golgi membranes at the beginning and the end of the 4-s tracking period. As shown in Figure 4D, no correlation between tubule length and the distance to Golgi membranes was found in control cells. In contrast, a negative correlation was found in cells treated with CK-666. Finally, we investigated whether Arp2/3 inhibition affects the stop-and-go movement characteristic of anterograde carriers moving to the Golgi apparatus [30,40,41]. The percentage of GFP-RAB1+ tubules undergoing pause events during the tracking period was found to be the same in DMSO and CK-666 treated cells (Figure 4G,I).

Altogether, the above results suggest that the long CK-666 induced GFP-RAB1+ tubules are less motile and less prone to reach the Golgi membranes than the shorter ones.

### 3.4. Arp2/3 Inhibition Decreases the Rate of Cargo Arrival to the Golgi Apparatus without Affecting the Early Stages of ER-to-Golgi Transport

**Figure 5:**
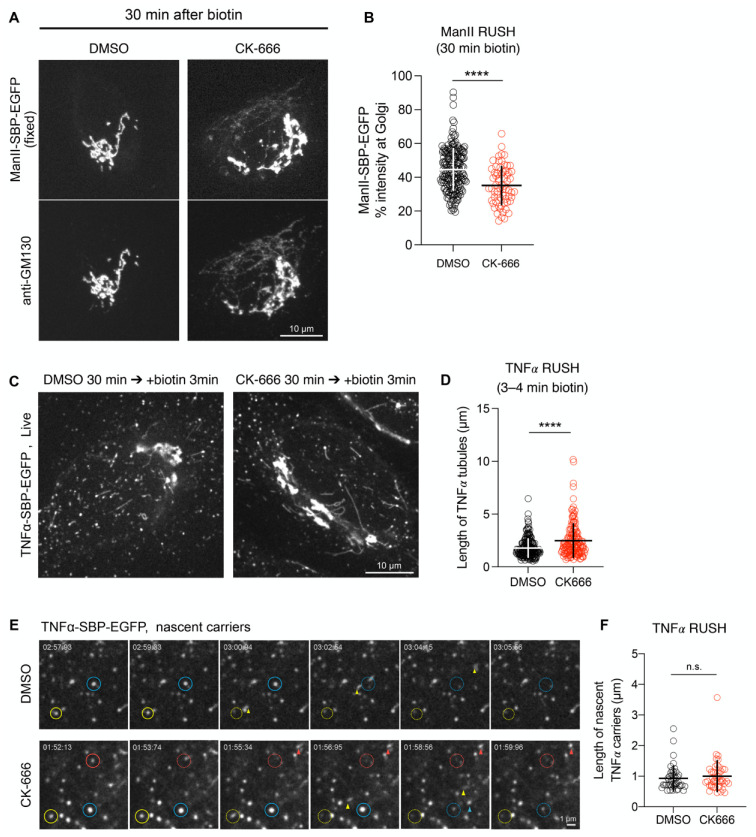
Arp2/3 inhibition impairs ER-to-Golgi transport. (**A**) HeLa cells expressing ManII-SBP-EGFP were incubated with CK-666 and biotin to induce cargo release; after 30 min, cells were fixed and immunolabeled; maximum projection of a confocal stack is shown. Scale bar: 10 µm. (**B**) Percentage of ManII-SBP-EGFP intensity in the Golgi at 30 min after biotin addition; CK-666 plot shows data of cells that contain long GM130+ tubules; raw data and mean ± SD from three independent experiments are indicated. (**C**) Representative images of time-lapse recordings of TNFα-SBP-EGFP expressing HeLa cells after biotin addition. Scale bar: 10 µm. (**D**) Length of TNFα- positive tubules quantified in time-lapse recordings of DMSO- or CK-666-treated cells after biotin addition; raw data and mean ± SD from three independent experiments are indicated. (**E**) Onset of directional movement of TNFα-positive elements captured by confocal microscopy; stationary TNFα+ punctae (circled) release nascent TNFα carriers (arrowheads); time after biotin addition is indicated in each frame. Scale bar: 1 µm. (**F**) Length of nascent TNFα+ carriers quantified in time-lapse recordings of DMSO- or CK-666-treated cells; raw data and mean ± SD from three independent experiments are indicated. n.s. not significant; **** *p* < 0.0001.

To investigate the effect of CK-666 on transport, we followed α-mannosidase II (ManII) coupled to GFP using the RUSH system, which allows to synchronize cargo exit from the ER [39]. As shown in Supplementary Video S6, the addition of biotin to the culture medium released GFP-ManII from the ER, which then reached the Golgi complex. In the majority of control cells, the GFP signal peaked at the Golgi within 30 min after cargo release. CK-666, added at the same time than biotin, induced the appearance in the Golgi region of tubular structures containing GFP-ManII and GM130 (Figure 5A). These tubules were then progressively incorporated into the Golgi membranes (Supplementary Video S6). The quantification 30 min after biotin addition of the GFP signal in the Golgi region of CK-666 treated cells showed that the drug inhibits by 25% the Golgi arrival of GFP-ManII (Figure 5B).

The length of transport carriers was studied using another cargo, TNF𝛼. As for ManII, CK-666 induced the appearance of long GFP-TNF𝛼 tubules in the Golgi region (Figure 5C,D; Supplementary Video S7). As previously described [28], RAB1 overlapped with the cargo-containing tubules (Supplementary Figure S3) indicating that RAB1 tubules correspond to transport intermediates moving from the ERES toward the Golgi. To investigate the effect of CK-666 at the early stages of ER-to-Golgi transport, cells were recorded at 0.2 s/frame after the addition of biotin. As shown in Supplementary Video S8, TNF𝛼 was observed in vesicles or very short tubules moving rapidly towards the cell center that likely represent nascent TNF𝛼 carriers. The addition of CK-666 did not significantly modify their morphology (Figure 5E,F), suggesting that the drug did not affect the early stage of ER-Golgi transport.

**Figure 6:**
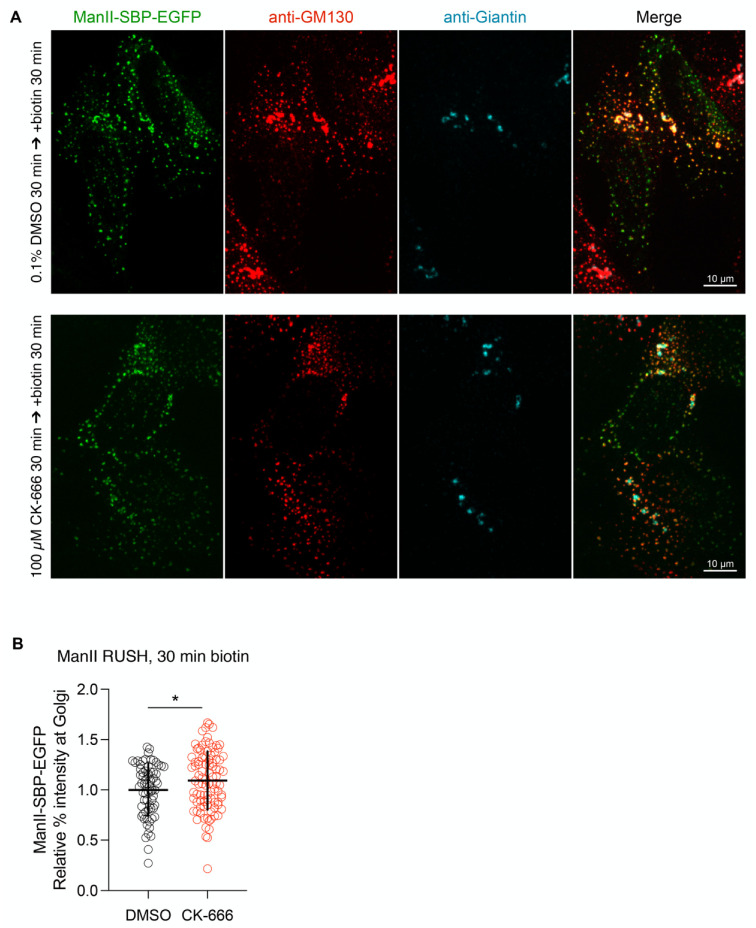
Arp2/3 inhibition does not affect ER-to-Golgi transport in the absence of microtubules. (**A**) HeLa cells expressing ManII-SBP-EGFP RUSH constructs were incubated in the presence of nocodazole at 4 °C, and then treated with DMSO or CK-666 prior to cargo release. 30 min after addition of biotin, cells were fixed and immunolabeled. Maximum projections of confocal stacks are shown. (**B**) Percentage of ManII-SBP-EGFP in the dispersed Golgi is shown as relative value. Raw data and mean ± SD from two independent experiments are indicated. * *p* < 0.05.

To further investigate the effect of Arp2/3 inhibition, we measured cargo transport in the absence of microtubules. Microtubules were depolymerized by incubating cells at 4 °C followed by nocodazole treatment [42]. In the absence of microtubules, Golgi membranes redistribute to ERES but remain competent for transport [43,44]. Accordingly, GFP-ManII can reach the redistributed Golgi elements following its release from the ER after biotin addition (Figure 6A). The incubation of nocodazole-treated cells with CK-666 did not affect ManII transport to the Golgi membranes, a slight increase in its transport kinetics being even observed (Figure 6B). In addition, no tubular elements containing GFP-ManII and stained for GM130 were observed.

### 3.5. Arp2/3 Inhibition Affects Acetylated Microtubule Network around the Golgi Apparatus

**Figure 7:**
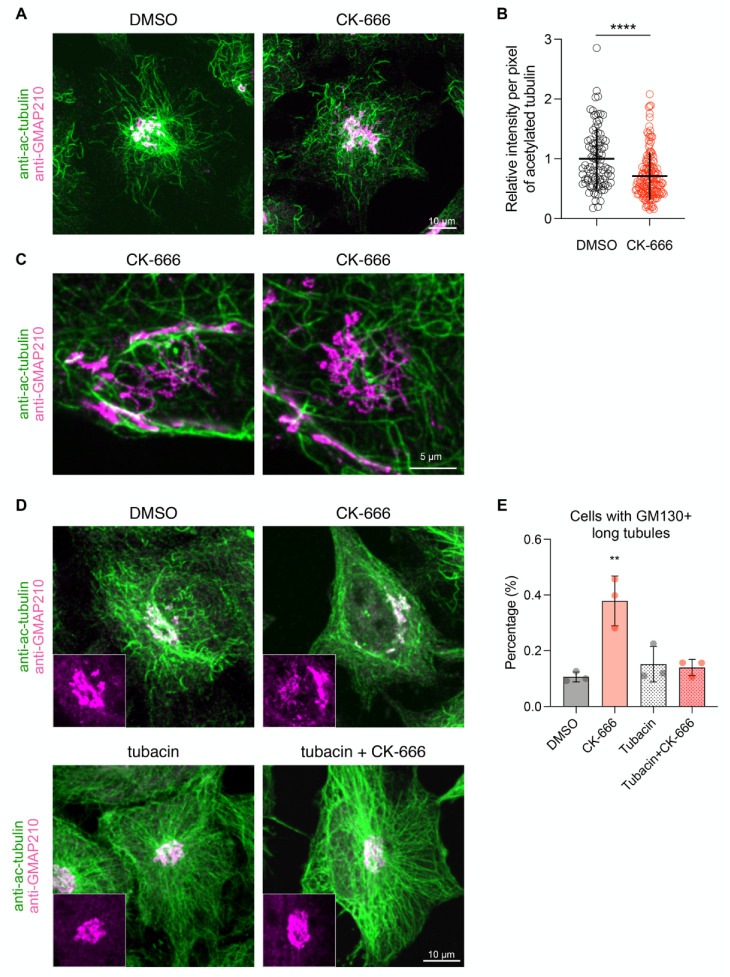
Arp2/3 inhibition induces loss of acetylated microtubules. (**A**) Maximum projection of confocal stacks of HeLa cells treated with DMSO or CK-666 immunolabeled with anti-acetylated tubulin and anti-GMAP210 antibodies; scale bar: 10 µm. (**B**) Total acetylated tubulin intensity normalized by cell area measured is shown as relative value to the mean value of the DMSO control. Raw data and mean ± SD from two independent experiments are indicated. (**C**) Confocal images of the centrosome and the Golgi region in CK-666-treated cells. Scale bar: 5 µm. (**D**) Maximum projection of confocal stacks of HeLa cells treated with DMSO, CK-666, tubacin or a mixture of both immunolabeled with anti-GM130 antibody. Inset shows GMAP210 signal at basal side of each cell. Scale bar: 10 µm. (**E**) Percentage of cells containing long GM130+ tubules. Raw data and mean ± SD from three independent experiments are indicated (DMSO, *n* = 298; CK-666, *n* = 415; tubacin, *n* = 312; tubacin + CK-666, *n* = 185 cells). ** *p* < 0.01; **** *p* < 0.0001.

Altogether, the above results suggest that CK-666 affects ER-to-Golgi trafficking around the Golgi in a microtubule-dependent manner. We thus investigated the organization of the microtubule network in the Golgi area of drug-treated cells. It is known that microtubules in this region are highly acetylated [45,46,47]. Consistently, we observed a dense network of acetylated microtubules around the Golgi in control cells. In contrast, this network was less dense in CK-666-treated cells (Figure 7A,C), reflecting a decrease in the total amount of acetylated tubulin (Figure 7B). It was recently reported that CK-666 induces histone deacetylase 6 (HDAC6)-dependent tubulin deacetylation, possibly by modulating the available pool of HDAC6 for tubulin [48]. We thus reasoned that the HDAC6 inhibitor tubacin could abolish the effect of CK-666 on membrane tubulation. This was indeed the case as no tubular structures positive for GM130 were observed in cells incubated with both tubacin and CK-666 that now showed dense and uniform acetylated microtubule throughout the cytoplasm (Figure 7D,E). This suggests that the accumulation of long RAB1/GM130+ tubules induced by CK-666 is directly linked to a decreased level of acetylated tubulin in the Golgi area.

## 4. Discussion

ER-to-Golgi transport is thought to occur in two main steps: the formation of membrane carriers, which repeatedly emerge at (or in the vicinity of) the ERES and move toward the IC/ERGIC compartment; and the movement of cargoes from the IC/ERGIC compartment to *cis*-Golgi membranes. Whether the IC/ERGIC is a stable or transient compartment is still a matter of debate [38]. We showed that CK-666 induced the tubulation of RAB1+ positive structures only in the vicinity of the Golgi complex. In addition, the drug does not alter the morphology of transport carriers containing TNF𝛼, which emerge from the ERES and continue to move towards the cell center. This suggests that unlike in the formation of transport carriers from Golgi/TGN or endosomal membranes [10,12,13,14,49,50,51,52], the actin cytoskeleton does not constitute a major driving force for membrane deformation in the early secretory pathway.

A main finding of this study is that acute Arp2/3 inhibition impacts the ER-Golgi transport through loss of acetylated microtubule network organized around the Golgi complex. The Golgi complex is a microtubule organizing center [46] which nucleates and stabilizes microtubules at both its *cis* and *trans* sides [47,53,54,55]. At TGN membranes, microtubules are nucleated via the microtubule plus-end tracking proteins, CLASPs and the golgin GCC185 [56]. At the *cis* side of the Golgi complex, the nucleation machinery involves AKAP450 and GM130 and loss of AKAP450 greatly reduces acetylated microtubules and impairs directional cell migration and ciliogenesis [47,57]. Unlike TGN-derived microtubules, which are connected to specific domains of the plasma membrane, including focal adhesions, and sustain polarized secretion of post-Golgi carriers [58,59], the role of *cis*-Golgi-nucleated microtubules in intracellular trafficking has not been established. Based on our results, we propose that acetylated microtubules assure transport efficiency in the late stages of ER-to-Golgi transport between IC/ERGIC and *cis*-Golgi membranes, and specifically between the pcIC and the Golgi apparatus. However, we do not exclude the possibility that retrograde transport between Golgi and ER is also perturbed by Arp2/3 inhibition. For instance, impaired recycling of components such as SNAREs required for the fusion of anterograde transport with *cis*-Golgi membranes could lead to the appearance of membrane tubules. Protein kinase A (PKA) activity was also shown to be required for fission and fusion of Golgi-to-ER retrograde tubules [60]. However, such tubules contain Giantin and Golgi resident enzymes, which was not observed following Arp2/3 inhibition.

Increased binding in vitro of kinesin-1/KIF5-B and dynein to acetylated microtubules has been documented [61,62]. In vivo, tubacin treatment of hippocampal neurons enhances anterograde transport to neurite tips of the kinesin-1 cargo protein JIP-1 [61]. In cortical neurons, microtubule acetylation stimulates both anterograde and retrograde transport of BDNF (brain-derived neurotrophic factor)-containing vesicles [62]. This suggests that in the vicinity of the Golgi complex, acetylated microtubules are preferentially used by transport vesicles moving toward both + and − ends of microtubules. Both kinesins (kinesin-1 and 2) and dynein are present at the IC/ERGIC [34,41,63,64,65]. Thus, a decrease in tubulin acetylation could thus affect bi-directional movement of RAB1+ positive carriers between IC/ERGIC and *cis*-Golgi membranes. Long-lived mechanically stable acetylated microtubules [66,67,68] may be important in sustaining stable transport tracks in the region where multiple transport pathways converge. Interestingly, we found that the speed of long RAB1+ tubules was significantly lower than that of short tubules. This suggest that the formation of membrane tubules could be the reflection of a traffic “jam” caused by the disappearance of acetylated microtubules preferentially used for transport. We provide evidence in this study that anterograde transport is affected; however, it remains to be investigated whether retrograde transport between Golgi and the ER is also affected.

The centrosome was recently shown to be able to promote Arp2/3-dependent actin filament assembly [69]. In addition, actin filaments can regulate microtubule growth at the centrosome [70]. This suggests that microtubule and actin assembly are functionally co-regulated in the pericentrosomal region of the cells. Our results illustrate that such an interplay between the actin and microtubule cytoskeleton is important for ensuring efficient cargo trafficking at the late stage of ER-to-Golgi transport.

## Data Availability

The data presented in this study are available on request from the corresponding author.

## Supplementary Materials

The following are available online at https://www.mdpi.com/article/10.3390/cells11010015/s1, Figure S1: Effects of CK-666, CK-689 and drug washout on GM130+ membranes.; Figure S2: The emergence of membrane tubules is related to centrosome position.; Figure S3: RAB1 overlaps with cargo-containing tubules.; Videos S1 and S2: Arp2/3 inhibition increases the length of GFP-RAB1+ tubules.; Video S3: Long GFP-RAB1+ tubules appear around the Golgi and the centrosome.; Video S4: Dynamic GFP-RAB1+ tubules aligned along microtubules.; Video S5: GFP-RAB1+ tubular structures disappear after CK-666 washout.; Video S6: ManII-SBP-EGFP release after biotin addition.; Video S7: TNFα-SBP-EGFP release after biotin addition.; Video S8: Movement of nascent TNFα carriers.
