# Supplementary material for: Branched Actin Maintains Acetylated Microtubule Network in the Early Secretory Pathway
Source: Cells. 2021 Dec 22;11(1):15. doi: 10.3390/cells11010015 (PMC8750537; doi:10.3390/cells11010015)
Supplement: Supplementary file 1 [file cells-11-00015-s001.zip › cells-1485344-supplementary.pdf]

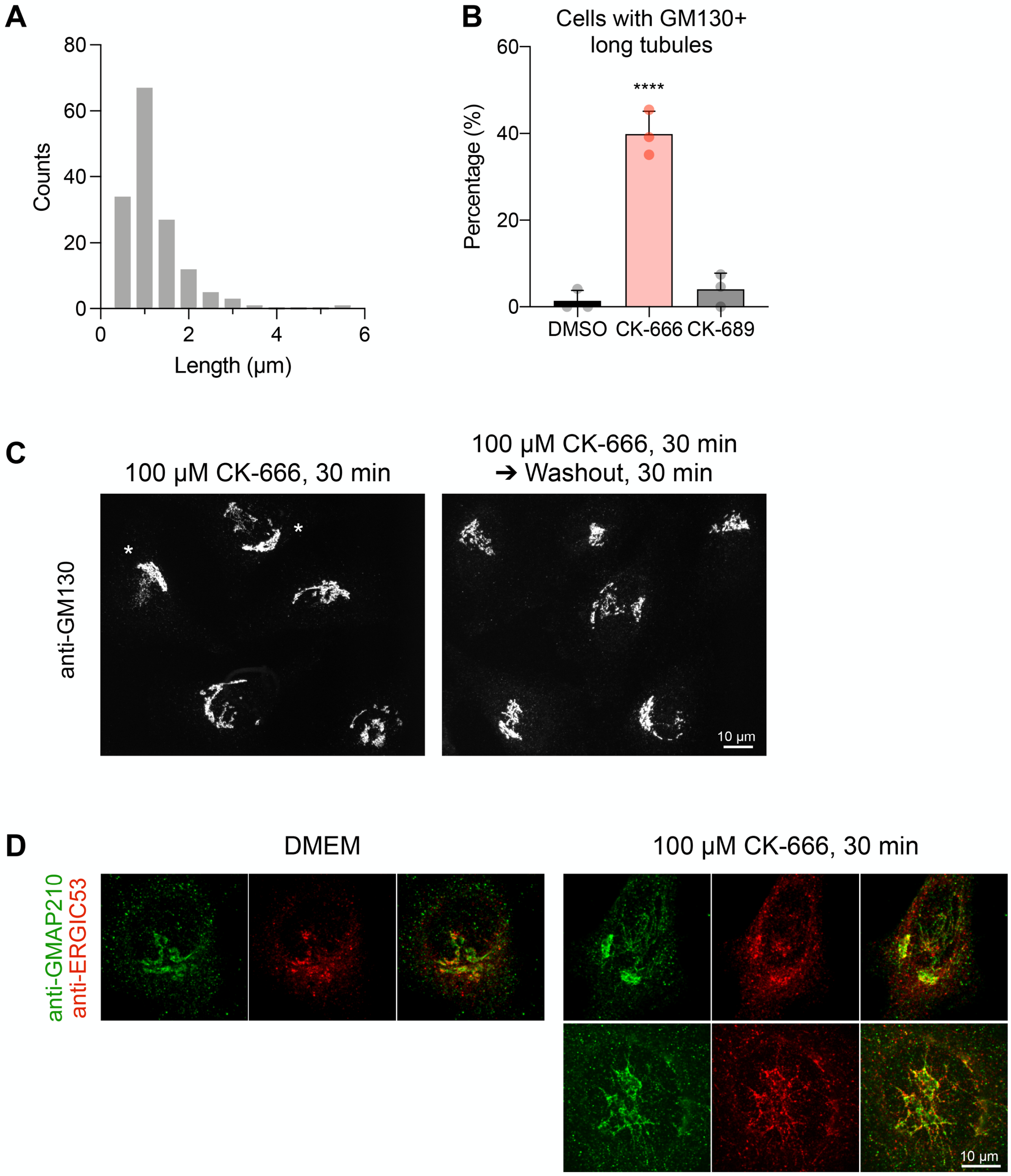

Figure S1. Effects of CK-666, CK-689 and drug washout on GM130+ membranes. (A) Histogram of the length of GM130-positive tubules quantified in DMSO-treated fixed cells. Mean length was 1.18 ± 0.67 µm and 95% percentile was 2.57 µm (n = 23 cells). (B) Percentage of cells containing long GM130-positive tubules in control (DMSO) and after treatment with CK-666 or CK-689. Raw data and mean ± SD are indicated (DMSO, n = 98; CK-666, n = 175; CK-689, n = 107). **** $p < 0.0001$. (C) Cells treated with CK-666 were fixed and immunolabelled for GM130 without (left) or after 30-min drug washout (right). Maximum projections of confocal slices around the Golgi apparatus are shown. Cells that contain long GM130+ tubules are indicated by

asterisks. Scale bar: 10 μm. (D) Control (DMEM) and CK-666-treated cells labelled with anti-ERGIC53 and anti-GMAP210 antibodies. Maximum projections of confocal slices around the Golgi apparatus are shown. Scale bar: 10 μm.

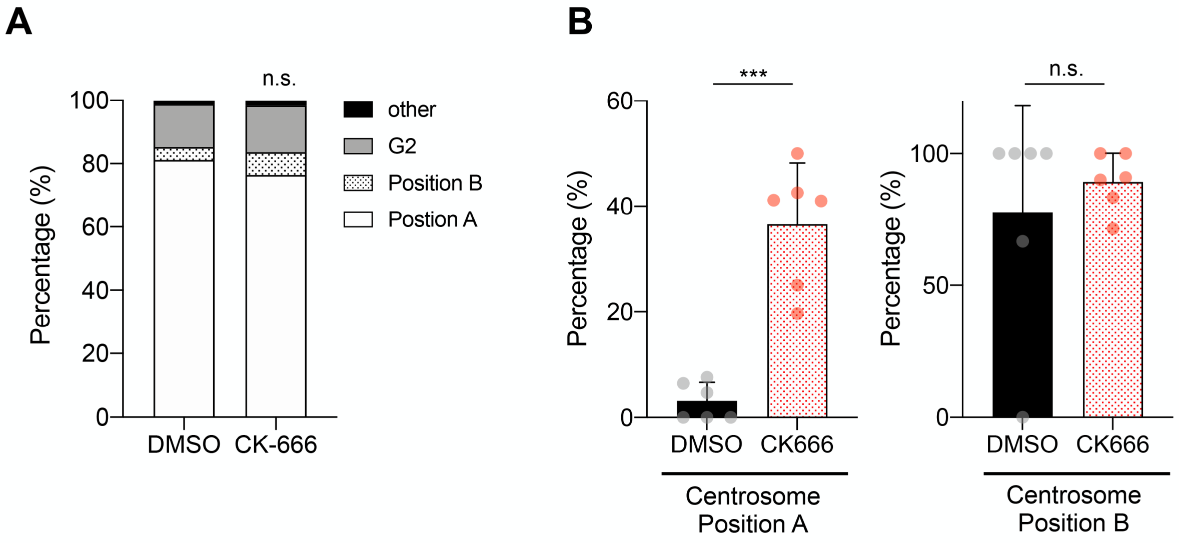

Figure S2. The emergence of membrane tubules is related to centrosome position. (A) Distribution of control (DMSO) and CK-666-treated cells within population A (centrosome close to Golgi membranes), population B (centrosome away from Golgi membranes), and in G2. Cells with undetectable centrosome were categorized as "other". Mean values from 6 independent experiments are shown. (B) Percentage of cells containing long GM130+ tubules in populations A and B. Raw data and mean ± SD are indicated (Population A: DMSO, n = 312; CK-666, n = 446. Population B: DMSO, n = 14; CK-666, n = 44). n.s. not significant; *** $p < 0.001$.

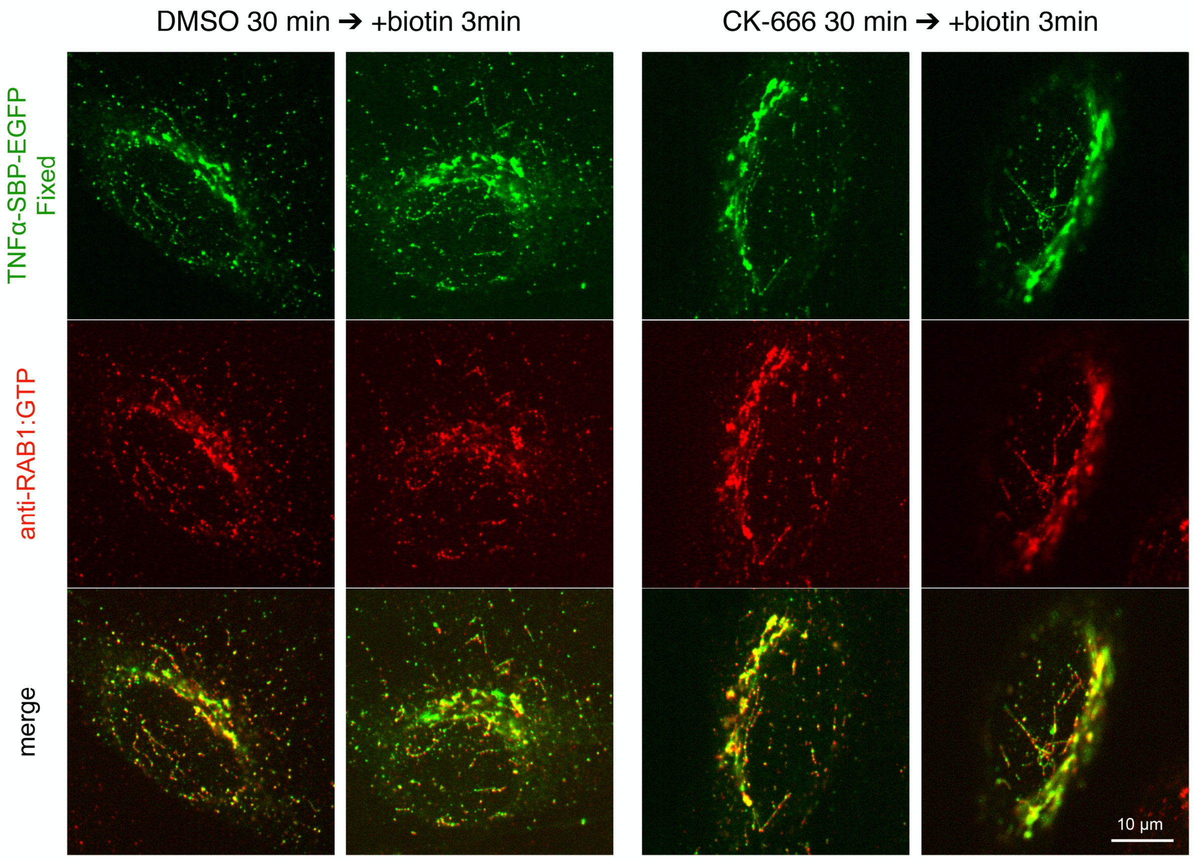

Figure S3. RAB1 overlaps with cargo-containing tubules. HeLa cells expressing TNFα -SBP-EGFP were incubated with DMSO or CK-666 for 30 min followed by biotin addition. After 3 min, cells were fixed and immunolabeled. Scale bar: 10 μm.

Videos S1, S2. Arp2/3 inhibition increases the length of GFP-RAB1+ tubules.
Time-lapse confocal imaging of HeLa cells expressing GFP-RAB1 at 0.2 s interval. GFP-RAB1 was monitored before and after the addition of CK-666, as described in Figure 3.

Video S3. Long GFP-RAB1+ tubules appear around the Golgi and the centrosome.
Time-lapse confocal imaging of HeLa cells expressing GFP-RAB1 and Centrin1-mCherry in the presence of CK-666.

Video S4. Dynamic GFP-RAB1+ tubules aligned along microtubules.
Time-lapse confocal imaging of HeLa cells expressing GFP-RAB1 and mCherry-tubulin in the presence of CK-666.

Video S5. GFP-RAB1+ tubular structures disappear after CK-666 washout.
Time-lapse confocal imaging of GFP-RAB1+ structures around the Golgi after CK-666 washout. Long tubules progressively disappeared.

Video S6. ManII-SBP-EGFP release after biotin addition.
ManII-SBP-EGFP release after biotin addition in the presence of DMSO (left) or CK-666 (right) shown by time-lapse confocal imaging.

Video S7. TNFα-SBP-EGFP release after biotin addition.
TNFα-SBP-EGFP released after biotin addition in the presence of DMSO (left) or CK-666 (right) shown by time-lapse confocal imaging.

Video S8. Movement of nascent TNFα carriers.
Time-lapse recordings of the onset of directional movement of nascent TNFα carriers shown in Figure 5E.
